# Novel genes involved in severe early-onset obesity revealed by rare copy number and sequence variants

**DOI:** 10.1371/journal.pgen.1006657

**Published:** 2017-05-10

**Authors:** Clara Serra-Juhé, Gabriel Á. Martos-Moreno, Francesc Bou de Pieri, Raquel Flores, Juan R. González, Benjamín Rodríguez-Santiago, Jesús Argente, Luis A. Pérez-Jurado

**Affiliations:** 1 Genetics Unit, Universitat Pompeu Fabra, Barcelona, Spain; 2 Hospital del Mar Research Institute (IMIM), Barcelona, Spain; 3 Centro de Investigación Biomédica en Red de Enfermedades Raras (CIBERER), Instituto de Salud Carlos III, Barcelona, Spain; 4 Departments of Pediatrics & Pediatric Endocrinology, Hospital Infantil Universitario Niño Jesús, Universidad Autónoma de Madrid, Madrid, Spain; 5 Hospital de la Princesa Research Institute, Madrid, Spain; 6 Centro de Investigación Biomédica en Red de fisiopatología de la obesidad y nutrición (CIBEROBN), Instituto de Salud Carlos III, Madrid, Spain; 7 Center for Research in Environmental Epidemiology (CREAL), Barcelona, Spain; 8 Centro de Investigación Biomédica en Red de Epidemiología y Salud Pública (CIBERESP), Instituto de Salud Carlos III, Barcelona, Spain; 9 Quantitative Genomic Medicine Laboratories (qGenomics), Esplugues de Llobregat, Spain; 10 IMDEA Food Institute, CEI UAM & CSIC, Madrid, Spain; Centre for Genomic Regulation (CRG), SPAIN

## Abstract

Obesity is a multifactorial disorder with high heritability (50–75%), which is probably higher in early-onset and severe cases. Although rare monogenic forms and several genes and regions of susceptibility, including copy number variants (CNVs), have been described, the genetic causes underlying the disease still remain largely unknown. We searched for rare CNVs (>100kb in size, altering genes and present in <1/2000 population controls) in 157 Spanish children with non-syndromic early-onset obesity (EOO: body mass index >3 standard deviations above the mean at <3 years of age) using SNP array molecular karyotypes. We then performed case control studies (480 EOO cases/480 non-obese controls) with the validated CNVs and rare sequence variants (RSVs) detected by targeted resequencing of selected CNV genes (n = 14), and also studied the inheritance patterns in available first-degree relatives. A higher burden of gain-type CNVs was detected in EOO cases versus controls (OR = 1.71, p-value = 0.0358). In addition to a gain of the *NPY* gene in a familial case with EOO and attention deficit hyperactivity disorder, likely pathogenic CNVs included gains of glutamate receptors (*GRIK1*, *GRM7*) and the X-linked gastrin-peptide receptor (*GRPR*), all inherited from obese parents. Putatively functional RSVs absent in controls were also identified in EOO cases at *NPY*, *GRIK1* and *GRPR*. A patient with a heterozygous deletion disrupting two contiguous and related genes, *SLCO4C1* and *SLCO6A1*, also had a missense RSV at *SLCO4C1* on the other allele, suggestive of a recessive model. The genes identified showed a clear enrichment of shared co-expression partners with known genes strongly related to obesity, reinforcing their role in the pathophysiology of the disease. Our data reveal a higher burden of rare CNVs and RSVs in several related genes in patients with EOO compared to controls, and implicate *NPY*, *GRPR*, two glutamate receptors and *SLCO4C1* in highly penetrant forms of familial obesity.

## Author summary

Although there is strong evidence for a high genetic component of obesity, the underlying genetic causes are largely unknown, mostly due to the highly heterogeneous nature of the disorder. In this work, we have focused on the most severe end of the spectrum, severe obesity with early-onset in childhood, which is more likely due to genetic alterations. We screened for rare copy number variation (CNV) a sample of 157 Spanish children with early-onset obesity using molecular karyotypes and then studied the genes altered by CNVs in 480 cases and 480 non-obese controls. We identified a higher burden of gain-type CNVs in cases as well as several CNVs and sequence variants that were specific of the obese population. Interestingly, the genes identified shared co-expression partners with known obesity genes. Among those, the genes encoding the neuropeptide Y (*NPY*), two glutamate receptors (*GRIK1*, *GRM7*), the X-linked gastrin-peptide receptor (*GRPR*), and the organic anion transporter (*SLCO4C1)* are novel obesity candidate genes that may contribute to highly penetrant forms of familial obesity.

## Introduction

Early-onset overweight (body mass index [BMI] ≥ 85th percentile for age and sex) and obesity (BMI ≥ 95th percentile for age and sex) currently affects 27.8% of children in Spain (Spanish National Health Survey, 2011–2012), being the most prevalent chronic disorder in childhood and adolescence. In the United States, 17.3% of children aged 2 to 19 years are obese, 5.9% meet criteria for class 2 obesity (BMI ≥ 120% of the 95th percentile or BMI ≥ 35), and 2.1% have class 3 obesity (BMI ≥ 140% of the 95th percentile or BMI ≥ 40) [1]. Early-onset obesity (EOO) entails several comorbidities and predisposes to obesity and related diseases during adulthood, being one of the most important health problems in developed countries.

Single gene alterations with Mendelian inheritance account for less than 5% of non-syndromic cases of severe EOO [2], including mutations in the *LEP* (MIM 164160) or *LEPR* (MIM 601007) genes [3–5], as well as in *MC4R* (MIM 155541) [6,7] which are the most common cause of monogenic obesity. Genetic, genomic and epigenetic alterations have also been identified in syndromic forms of obesity, such as Bardet-Biedl syndrome (MIM 209900) [8], Prader-Willi syndrome (MIM 176270) [9], Beckwith-Wiedemann syndrome (MIM 130650) [10] and other rare diseases. However, obesity is generally considered a multifactorial disorder with high heritability (50–75%), probably higher in early-onset cases [11]. To date multiple studies have tried to elucidate genetic factors contributing to the etiopathogenesis of obesity, and relevant SNPs in more than 100 loci have been identified by Genome Wide Association Studies (GWAS), including those near genes such as *FTO* (MIM 610966), *MC4R*, *NEGR1* (MIM 613173) or *TMEM18* (MIM 613220) [12–15]. Nevertheless, the fraction of BMI variance explained by these GWAS top hits is estimated to be only around 2% [16]. Even the infinitesimal model, that combines the effect of all common autosomal SNPs, only explains ∼17% of the variance in BMI [17]. Gene-based meta-analysis of GWAS allowed the identification of regions with high allelic heterogeneity and new loci involved in obesity [18,19].

In addition, several common and rare copy number variants (CNV) contributing to the heritability of BMI and obesity have been reported, including deletions upstream of the *NEGR1* gene [13], proximal and distal deletions at 16p11.2 [20], gains at 10q26.6 containing the *CYP2E1* gene (MIM 124040) [21], and homozygous deletions at 11q11 encompassing olfactory receptor genes [22], among others. While several studies in large datasets led to the conclusion that common CNVs are not a major contributor [22], a significantly increased burden of rare CNVs was documented in cases of severe obesity with and without associated developmental delay [15,23]. Specifically, a significant enrichment for CNVs larger than 100 Kb and with a population frequency lower than 1% was identified in subjects with isolated severe EOO when compared to controls [15].

In the present study, we have analyzed the contribution to the phenotype of rare and common CNVs as well rare sequence variants (RSVs) in CNV-related genes in a large Spanish sample of patients with isolated severe EOO using case-control and family-based approaches, with the goal to identify novel genes involved in the pathophysiology of severe obesity.

## Results

**Fig 1:**
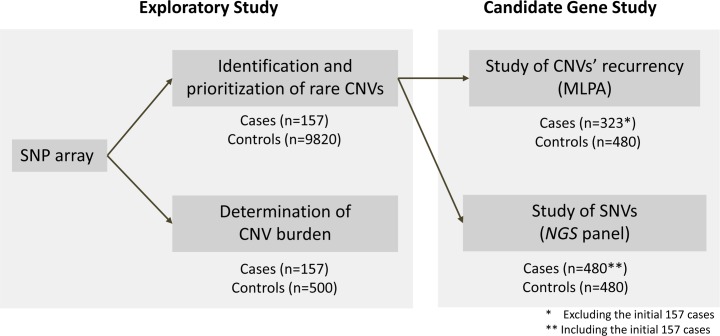
Workflow of the approach followed to study CNVs and RSVs in a subset of selected genes. The different control cohorts used for each of the analysis are shown, in addition to the number of obese patients and controls studied in each step.

We used a sequential strategy to identify genes potentially related to EOO through the analysis of CNVs by molecular karyotyping and subsequent mutation screening using a DNA pooled approach in a subset of selected genes. The strategy, including the samples used for each step, is summarized in Fig 1.

### CNV burden in EOO

A total of 42 autosomal CNVs fulfilling the established criteria (>100 kb, gene containing and present in <1/2000 population individuals) were identified in 36 cases (22.9%). We detected 7 deletions and 35 gains (100.1–3,590kb in length), with 5 samples harboring more than one rearrangement (Table 1). MLPA was used for validation (42/42) and determination of the inheritance pattern (41/42): all tested rearrangements were inherited except for the larger deletion. We also detected and validated 2 additional CNVs on the X-chromosome for a total of 44 CNVs.

**Table 1:** Summary of copy number variations detected in 157 samples of patients with severe obesity. Control frequency refers to the frequency of the same type of rearrangement, deletion or duplication, in the control cohort (9,820 subjects). Progenitor phenotype refers only to the progenitor carrying the alteration. Hg19 assembly. F: female; M: male; Mat: maternal; Pat: paternal; N: normal; OW: overweight; OB: obesity; NA: not available; +: description of a mouse model with a phenotype related to body mass index (S1 Table). The genes interrupted at any of the breakpoints of the CNV are shown in **boldface**.

Case	Gender	Gain/ Loss	Region	Length (kb)	Hg19 coordinates	Genes	Inheritance	Progenitor phenotype	Control frequency	Mouse model
**Recurrent alterations in obese patients**										
**Ob_1**	F	Gain	9q34.3	149	chr9:138149166–138298164	*C9orf62*	Mat	N	1	
**Ob_2**	F	Gain	9q34.3	139	chr9:138149166–138288052	*C9orf62*	Pat	OW	1	
**Ob_3**	F	Gain	9q34.3	139	chr9:138149166–138288052	*C9orf62*	Mat	OW	1	
**Ob_4**	M	Gain	7p22.1	106	chr7:5785086–5891221	***RNF216***,***ZNF815***	Mat	OB	4	
**Ob_5**	F	Gain	7p22.1	106	chr7:5785086–5891221	***RNF216***,***ZNF815***	Pat	OB	4	
**Ob_6**	F	Loss	11p15.4	104	chr11:4583029–4687238	*TRIM68* and 5 more genes	Pat	OB	2	
**Obese specific alterations, not found in 10,320 controls, co-segregating with the phenotype in the family**										
**Ob_7**	F	Gain	3q29	1169	chr3:196533320–197701913	*DLG1*,***PAK2***,***LMLN*** and 10 more genes	Mat	OB	0	+
**Ob_8**	F	Gain	4q12	361	chr4:53842714–54203701	***SCFD2***	Pat	OB	0	
**Ob_9**	F	Loss	6q23.2	166	chr6:133386327–133552737	*LINC00326*	Mat	OB	0	
**Ob_10**	M	Loss	5q21.1	157	chr5:101620174–101776835	***SLCO4C1***,***SLCO6A1***	Pat	OB	0	
**Ob_11**	M	Gain	7p15.3	202	chr7:21858215–22059791	*CDCA7L*,***DNAH11***	Pat	OB	0	
**Ob_12**	M	Gain	7p15.3	137	chr7:24258773–24395900	*NPY*	Mat	OB	0	+
**Ob_13**	F	Gain	10p14	1029	chr10:10743956–11773389	*CELF2* and 4 more genes	Mat	OB	0	
**Ob_14**	M	Gain	14q31.1	105	chr14:81204951–81309536	***CEP128***	Mat	OB	0	
**Ob_15**	F	Gain	14q31.3	274	chr14:89171711–89445350	***EML5***,*TTC8*	Mat	OB	0	+
**Ob_16**	M	Gain	19p13.3	428	chr19:2221792–2650034	*AMH*,***DOT1L***,***GNG7*** and 14 more genes	Pat	OB	0	
**Ob_17**	M	Gain	20p12.1	220	chr20:13255679–13476090	***ISM1***,***TASP1***	Pat	OB	0	+
**Copy number variations detected in single cases**										
**Ob_18**	F	Gain	1p36.13	223	chr1:17202355–17425829	*ATP13A2*,***PADI2*** and 3 more genes	Mat	N	0	
**Ob_19**	M	Gain	1p31.3	405	chr1:61704166–62109502	***NFIA***	Mat	OW	3	
**Ob_20**	M	Gain	2q24.1	336	chr2:159176303–159512667	***CCDC148***,***PKP4***	Mat	N	0	
**Ob_21**	M	Gain	3p26.1	214	chr3:4272253–4486303	*SETMAR*,***SUMF1***	Pat	N	2	
**Ob_22**	M	Gain	3p26.1	364	chr3:7660133–8024019	***GRM7***	Mat	OW	3	
**Ob_15**	F	Loss	3q12.3	3590	chr3:101816344–105406145	*ZPLD1*, *ALCAM*, ***CBLB***	*De novo*	-	0	
**Ob_23**	M	Gain	4q13.3	101	chr4:71055318–71156301	*C4orf7*,*CSN3*,*ODAM*	Mat	N	0	
**Ob_18**	F	Gain	5p15.33	295	chr5:303686–598237	***AHRR*,*PDCD6*,** *and 6 more*	Mat	OW	3	
**Ob_24**	M	Gain	5q35.3	397	chr5:179220638–179617799	*C5orf45*,***RASGEF1C*** *and 6 more*	Pat	N	0	
**Ob_25**	M	Gain	6q15	445	chr6:89349438–89793993	***PNRC1***,***RNGTT***	Mat	OW	0	
**Ob_26**	F	Gain	7p14.1	105	chr7:40117098–40221714	***C7orf10***,*C7orf11*,***CDK13***	Pat	OB	1	
**Ob_27**	F	Gain	9p24.3	448	chr9:396232–844001	***DMRT1***,***DOCK8***,*KANK1*	Mat	N	2	
**Ob_15**	F	Loss	10q21.3	156	chr10:69418270–69574169	***CTNNA3***,***DNAJC12***	Mat	OB	1	
**Ob_28**	M	Gain	11p13	129	chr11:32986850–33116054	***CSTF3*,*QSER1*** and 3 more genes	Pat	N	0	
**Ob_29**	F	Gain	12q23.3	116	chr12:104476277–104591886	***HCFC2***,*NFYB*	Pat	NA	0	
**Ob_30**	M	Gain	12q24.33	218	chr12:131620578–131838842	***GPR133***,*LOC116437*	Pat	OB	1	
**Ob_16**	M	Gain	15q25.2	418	chr15:84414592–84832932	***ADAMTSL3***,*EFTUD1P1*	Pat	OB	1	
**Ob_31**	M	Gain	17q23.1	248	chr17:58113570–58361461	*CA4*,***USP32*** and 5 more genes	NA	NA	0	
**Ob_32**	F	Gain	18p11.31	245	chr18:6308208–6553040	***L3MBTL4***,***LOC100130480***,*MIR4317*	Mat	OW	0	
**Ob_5**	F	Gain	18p11.21	262	chr18:12917703–13180058	*CEP192*,*SEH1L*	Mat	N	2	
**Ob_14**	M	Gain	20p12.1	204	chr20:13867165–14070869	***MACROD2***,***SEL1L2***	Pat	N	0	
**Ob_33**	F	Gain	21q21.3	163	chr21:31280603–31443375	***GRIK1***	Pat	OB	1	+
**Ob_34**	F	Loss	22q11.22	261	chr22:22312292–22573637	***TOP3B***	Pat	OB	2	
**Ob_35**	F	Loss	22q11.22	676	chr22:22981587–23657613	***BCR*** *and 7 more*	Mat	NA	0	
**Ob_36**	M	Gain	22q13.33	198	chr22:50342728–50584201	*IL17REL*,*MLC1*,***MOV10L1***,*PIM3*	Pat	NA	0	
**Copy number variations detected in X chromosome**										
**Ob_37**	F	Gain	Xp22.2	305	chrX:15952591–16257827	*GRPR*	Mat	N	*1*	+
**Ob_6**	F	Gain	Xq26.2	369	chrX:130639755–131008470	*LOC286467*,*OR13H1*,***IGSF1***	Pat	OB	*0*	

Clinical data about the parental phenotype was available in all but two families with CNVs. The progenitor harboring the CNV was obese (defined as BMI >30) in 21 cases (53.8%), was overweight (BMI 25–30) in 7 cases (17.9%) and had a BMI in the normal range in 11 cases (28.2%). More than half of the rare CNVs (25 of 44), 21 gains and 4 deletions, were not found in any of the 9,820 adult population controls.

In order to analyze the global burden of rare CNVs in EOO, we compared the amount, type and length of autosomal CNVs in patients (157) with respect to 500 Spanish population controls (Table 2). Rare CNVs were found in 15.8% of controls with respect to the 22.9% frequency found in patients (p = 0.053). Rare CNVs were predominantly gains in both cohorts (83.3% in EOO patients and 77.6% in controls). When the frequency of deletions and gains was analyzed separately, no differences were observed in deletion-type CNVs (3.8% in controls and 4.5% in patients), while a statistically significant difference in the frequency of gains was detected (p = 0.0358). Thus, there is a higher burden of CNVs in EOO patients due to rare gain-type CNVs.

**Table 2:** Comparisons of the frequency of rare copy number changes in autosomal chromosomes >100kb detected in the patient cohort and in the cohort of 500 controls. In brackets the proportion of samples with the CNV and the proportion of the specific type of rearrangement.

Group	Alterations	Deletions	Duplications	Double hit	Samples
**Controls** **(500)**	85	19 (3.8% / 22.4%)	66 (12.6% / 77.6%)	5 (1.0%)	79 (15.8%)
**Obese patients (157)**	42	7 (4.5% / 16.7%)	35* (19.7% / 83.3%)	5 (3.2%)	36 (22.9%)

* p-value<0.05

If we consider specific CNVs as those not described in the initial 9,820 subjects used to establish the frequency of each alteration in the population, 8.2% control individuals carried a CNV fulfilling this criteria while the frequency was 14.0% in EOO patients, with this difference being statistically significant (p = 0.0422).

Regarding the co-occurrence of more than one CNV in the same subject, two or three hits were present in 3.2% patients and 1% controls. This difference was not statistically significant (p = 0.0644) likely due to the small sample size. The inheritance pattern of these alterations was established in patients; in two cases each alteration was inherited from a different parent and in the remaining three both rearrangements were inherited from the same progenitor. Case Ob_15 presented a third *de novo* event additionally to the two rearrangements inherited from her obese mother.

### Potentially pathogenic CNVs

CNVs were considered to have a higher probability to be pathogenic when they were exclusive of the EOO population, co-segregated with the phenotype in the family, disrupted known genes for the disorder and/or were found in more than one case.

**Fig 2:**
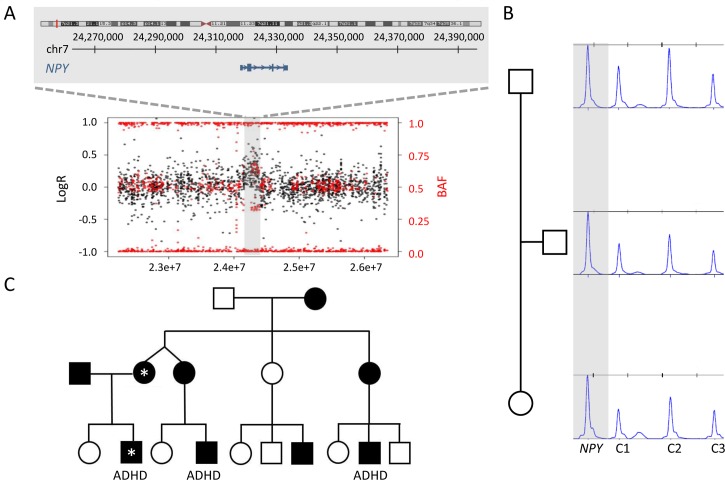
Detection, validation and inheritance of the duplication encompassing *NPY* in case Ob_12 and his family. A: Ideogram showing the location of the CNV and the specific genomic interval included in the duplication. The plot shows the results of SNP array with the Log R Ratio represented by black dots and the B Allele Frequency (BAF) represented by red dots. Hg19 assembly. B: MLPA of the trio showing the maternal inheritance of the rearrangement represented by a single probe (indicated). C: Pedigree of the family showing several cases with severe obesity, as well as ADHD (attention-deficit/hyperactivity disorder). The two individuals carrying the duplication are labeled by an *; samples from additional relatives were not available.

Nine duplications and two deletions were absent in 9,820 population controls and co-segregated with the phenotype in the family (Table 1). One of them was a 137kb gain in 7p15.3 containing a single coding gene, *NPY* (MIM 162640), identified in a male case (Ob_12) presenting with EOO and attention deficit hyperactivity disorder (ADHD) (Fig 2A). The CNV was inherited from the also obese mother (Fig 2B). Additional cases of severe EOO and ADHD were identified in the maternal branch of this family by report (Fig 2C), but unfortunately no additional samples or clinical data could be obtained.

Some CNVs overlapped with previously described microdeletion/microduplication syndromes (Table 1). Rearrangements partially overlapping with the critical region of the 22q11.2 distal deletion syndrome were identified in two patients. Ob_35 carried a 676kb deletion encompassing several genes including *RSPH14* (MIM 605663) and *GNAZ* (MIM 139160), while a more proximal deletion including *TOP3B* (MIM 603582) was detected in Ob_34. A gain of 348kb at 1q21.1 overlapping with the region of Thrombocytopenia-Absent Radius syndrome (MIM 274000) was detected in case Ob_39; as its frequency in controls was 1/1,720 it was not included in the subset of selected CNVs.

Two CNVs fulfilling the established criteria were identified in more than one patient (Table 1). A gain of 139kb in 9q34.3 only including *C9orf62* was found in three cases (Ob_1, Ob_2, Ob_3). However, the parents also carrying the CNV had either overweight or normal weight. Another gain of 106kb in 7p22.1 encompassing *RNF216* (MIM 609948) and *ZNF815P* was identified in two cases (Ob_4, Ob_5), inherited from obese parents. We then completed the analysis of the CNVs identified in the entire sample of obese individuals (n = 480) and the Spanish adult non-obese controls (n = 480) by MLPA. All rare CNVs were patient-specific except for a second patient with a deletion at 11p15.4. None of the rare CNVs were identified among controls except for the 106kb gain at 7p22.1 that was found in 5 controls. The re-analysis of SNP array data unraveled the complexity of mapping this region due to small segmental duplications and was used to determine the real frequency of the rearrangement, which was above the established threshold of the study (1/2,000).

### Association study with common CNVs

We also explored more common CNVs already described in association with obesity. The gain in 10q26.3 including *CYP2E1* was more common in patients than in controls (6.4% vs 3.6%) as previously described [21], but did not reach significance (OR: 2.01, CI95% 0.93–4.36, p-value = 0.075). The frequency of the homozygous deletion encompassing olfactory receptors in 11q11 was 5.1% in cases, which was slightly lower than the frequency in the control cohort (6.7%). Therefore, our data did not replicate the previous findings that indicate a preferable transmission of the 11q11 deletion to obese children [22]. In addition, in this study we did not detect alterations in the 16p11.2 region, including or next to the *SH2B1* (MIM 608937) gene.

### Identification of RSVs in novel genes by targeted capture sequencing of pooled DNA

All genes included and/or disrupted by CNVs found in more than one patient and/or co-segregating in a familial case were selected for sequence analysis (n = 14): *SCFD2*, *NPY*, *ISM1* (MIM 615793), *TASP1* (MIM 608270), *GRM7*, *LOC401164*, *TRIML1*, *SLCO4C1*, *SLCO6A1*, *C11orf40*, *TRIM68* (MIM 613184), *GRIK1*, *TOP3B* and *GRPR*. In order to sequence the total number of patients (480) and controls (480) in a cost-efficient manner, pools of 20 DNA samples were sequenced with each DNA sample located in two pools.

We first validated the suitability and specificity of the pipeline to detect real variants among the pools. RSVs were considered when they had a frequency below 1/1.000 in the public database of the Exome Sequencing Consortium (ExAC) representing more than 60,000 exomes [24]. We selected 23 alterations predicted to be in a single sample and reanalyzed the same sample by Sanger sequencing. All 23 RSVs were validated in the specific samples.

We then compared the total burden of RSVs per gene between patients and controls. Significant differences were identified in a few loci, namely *NPY*, *GRIK1* (MIM 138245) and *GRPR* (MIM 305670) (Table 3). A single missense RSV in *NPY* (p.V86D) was identified in patient Ob_158, while no RSVs of this gene were found in controls. Although the residue is not evolutionarily conserved and is located outside the main functional domain, the change is likely to affect the shape and the affinity of the NPY protein and has not been described in ExAC. The study of parental samples revealed that the RSV was inherited from the obese father (BMI 34.3 kg/m^2^). The low frequency of missense variants in this gene in the ExAC database (only 27 among 118.884 alleles) further reinforces its functional relevance.

**Table 3:** Point mutations detected by pooled DNA sequencing in the cohort of 480 patients. Control frequency refers to the allele frequency of the same variant in the subjects included in the ExAC database (60,706 unrelated individuals). Progenitor phenotype refers only to the progenitor carrying the alteration. Hg19 assembly. F: female; M: male; Mat: maternal; Pat: paternal; NA: not available.

Gene name	Variant	cDNA level	Protein level	Phylo P	Control frequency	Case	Gender	Inheritance	Progenitor phenotype
*NPY*	chr7:24329186	c.T257A	p.V86D	0.798641	0	Ob_158	F	Pat	Obesity
*GRPR*	chrX:16142093	c.G17C	p.C6S	0.992202	0.00095	Ob_159	F	NA	NA
*GRPR*	chrX:16142335	c.C259A	p.L87M	0.948124	0.00097	Ob_160	M	Mat	Obesity
*GRPR*	chrX:16170380	c.T767C	p.I256T	0.997201	0	Ob_161	F	NA	NA
*GRPR*	chrX:16168672	c.G658A	p.V220I	0.787201	0.00049	Ob_38	F	NA	NA
						Ob_162	M	Mat	Normal
*GRIK1*	chr21:30909580	c.C2689T	p.R897X	0.999731	0.00006	Ob_163	M	NA	NA
*SLCO4C1*	chr5:101606433	c.A697C	p.I233L	0.974709	0	Ob_10	M	Mat	Normal

A nonsense mutation (p.R897X) was identified in *GRIK1*, encoding the ionotropic glutamate receptor 1, in patient Ob_163. This nonsense variant has a frequency below 1/15000 alleles and, generally, nonsense and frameshift variants at the *GRIK1* gene are rare, representing less than 1/3000 alleles in the ExAC database. Additional missense mutations were identified in both glutamate receptors (*GRIK1* and *GRM7* (MIM 604101)), but with no significant differences between cases and controls.

Four different missense mutations were detected in *GRPR* in five patients with obesity, while no mutations in this gene were found in controls (5/662 alleles vs 0/726 alleles; p = 0.0245). One of the mutations has never been found in ExAC, while the remaining three had frequencies <1/1000 alleles and all are predicted to result in significant functional consequences.

Finally, a RSV in *SLCO4C1* (MIM 609013) was identified in a patient harboring a deletion encompassing the same gene previously identified by CMA. The RSV (p.I233L) has not been described previously and affects a highly conserved amino acid (phylo P = 0.975). The frequency of the deletion in the control cohort is 0/9,820. Parental studies confirmed that each progenitor had transmitted one of the alterations; the deletion was inherited from the obese father and the RSV from the non-obese mother. These findings are compatible with a recessive pattern of inheritance or a two-hit mechanism, with a major contribution of the CNV (inherited from an obese progenitor) and an additional and milder effect of the RSVs (inherited from a non-obese progenitor).

### Co-expression enrichment analyses

**Fig 3:**
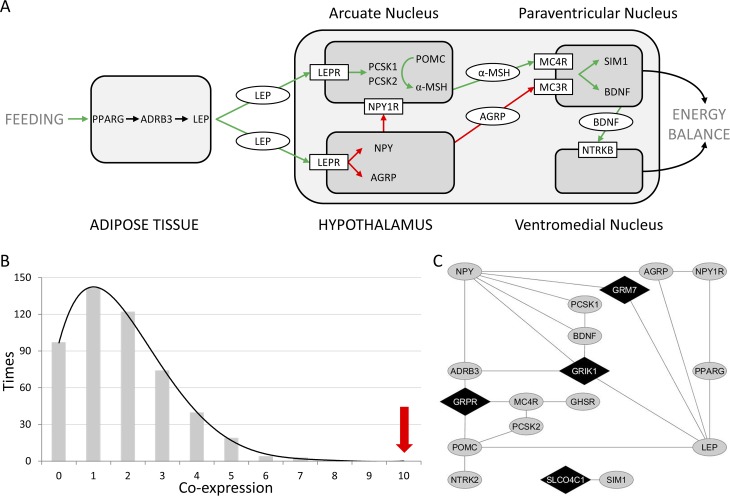
Enrichment analyses of shared co-expressed partners between 14 known obesity-related genes and the candidate genes from this study. A: Key players on the regulation of food intake and energy expenditure mostly act through the leptin-melanocortin pathway. B: Shared co-expressed partners between the four candidate genes (*GRIK1*, *GRM7*, *GRPR*, *SLCO4C1*) and 500 randomly generated gene sets. The number shared co-expressed partners of the candidate genes with the 14 obesity-related genes are indicated by a red arrow. C: Co-expression network including the selected obesity-related genes and *GRIK1*, *GRM7*, *GRPR*, *SLCO4C1*. This network was visualized using Cytoscape.

We focused our subsequent analysis on four novel candidate genes considering our CNVs and RSVs findings: *GRIK1*, *GRM7*, *GRPR* and *SLCO4C1*. To explore their possible role in obesity, we looked for co-expressions with a stringent list of 15 genes previously related with obesity. We selected a total 10 genes with described highly penetrant mutations in severely obese patients, all of them coding for proteins of the leptin-melanocortin pathway: *LEP*[4], *LEPR*[5], *MC4R*[6], *POMC* (MIM 176830) [25], *PCSK1* (MIM 162150) [26], *MC3R* (MIM 155540) [27,28], *BDNF* (MIM 113505) [29], *NTRK2* (MIM600456) [30], *PPARG* (MIM 601487) [31] and *SIM1*(MIM 603128) [20,32]. We also included 5 additional genes with a relevant intermediary role in the same pathway: *ADRB3* (MIM 109691) [33], *PCSK2* (MIM 162151) [34,35], *NPY*[36], *NPY1R* (MIM 162641) [37], *AGRP* (MIM 602311) [36,38] (Fig 3A).

A total of 10 shared co-expressed partners were identified in the analysis between our 4 novel strongest candidate genes (*GRIK1*, *GRM7*, *GRPR* and *SLCO4C1*) and the set of 15 obesity-related genes described in the literature. The maximum number of shared co-expressed partners between our 4 genes and 500 genes sets randomly selected was 7, being the empirical p-value of this difference 0.002 (Fig 3B and 3C).

## Discussion

Our results reveal a relevant contribution of rare CNVs to the etiology of severe EOO with a significantly higher burden of gain-type CNVs in patients compared to controls (p = 0.0358). Among relatively common CNVs we only detected a non-significant higher frequency of the gain in 10q26.3 containing *CYP2E1* [21]. Previous studies reported a higher frequency of deletion-type CNVs in patients with severe EOO with and without developmental delay [23]. The sample studied here was stringently selected based on clinical exam and targeted genetic testing in order to exclude subjects with syndromic obesity. Thus, all patients presented isolated EOO without comorbid phenotypes such as developmental delay. This difference in the range of phenotype severity could explain the difference in the type of rearrangements found enriched in these cohorts. Although only one of the CNVs had occurred *de novo*, the progenitor carrying the alteration also presented overweight or obesity in 71.8% of cases, reinforcing the potential role of many of these genetic alterations in the pathophysiology of the disorder. The only *de novo* alteration (a 3.6Mb deletion encompassing only 3 genes) was detected in a girl with two additional rearrangements inherited from her obese mother.

We have also screened for point mutations using DNA pools in a subset of selected genes located in obese-specific CNVs [39]. Interestingly, we found additional RSVs in patients in 4 of the selected genes (*NPY*, *GRPR*, SLCO4C1 and *GRIK1)* reinforcing their putative role in the pathophysiology of obesity. Although the pooled DNA strategy might have some limitations such as underdetection of relatively common variants, the complete validation rate (100%) demonstrates its high specificity.

A maternally inherited gain in 7p15.3 only encompassing the *NPY* gene was identified in a patient with EOO and ADHD. The mother also presented severe obesity, as did several relatives from the maternal branch including two male cousins with associated ADHD. Additionally, a missense RSV also inherited from an obese progenitor was identified in another patient, while no alterations were identified in controls (480). A larger gain of approximately 3Mb on chromosome 7p15.2–15.3 encompassing NPY and other genes was previously described in all affected individuals of an extended pedigree presenting ADHD, increased BMI, and elevated NPY levels in blood [40]. Therefore, the gain encompassing only the *NPY* gene in patient Ob_12 and his obese mother, the point mutation in patient Ob_158 and her obese father while only 27 missense variants have been described among 118.884 alleles in ExAC are strong evidences supporting that gain of function mutations of *NPY* can cause severe obesity and ADHD.

NPY is a hypothalamic orexigenic peptide with neuromodulator functions in the control of energy balance and food intake. NPY is overproduced in the hypothalamus of leptin deficient ob/ob mice [41]; when depleted by genetic manipulation, ob/ob mice showed reduced food intake, increased energy expenditure and less obesity [42]. On the other hand, overexpression of NPY in noradrenergic neurons caused diet- and stress-induced gain in fat mass in a gene-dose-dependent fashion [43].

In humans, despite some conflictive reports, *NPY* gene variants have been significantly associated with weight changes from young adulthood to middle age and with risk of obesity [44]. NPY is widely expressed throughout the central nervous system (CNS) and a systematic review and meta-analyses of drug naïve case-control studies also suggested its implication in ADHD [45]. In addition, increased central availability of NPY by intracerebroventricular administration in male rats resulted in a shift of metabolism towards lipid storage and increased carbohydrate use, along with enhanced locomotor activity and body temperature [46].

Among other genes altered by the CNVs identified, we considered as probably pathogenic those exclusive of the EOO population that also presented exclusive RSVs co-segregating with the phenotype in the family. To further assess the possible implication of these strong candidate genes (*GRIK1*, *GRM7*, *GRPR* and *SLCO4C1*), we determined the co-expression patterns between them and 15 well-defined genes from the leptin-melanocortin pathway previously related to obesity. This analysis consistently identified a significant enrichment of co-expression shared partners among our genes and the subset of obesity related genes when compared to 500 randomly generated gene sets, reinforcing the possible role of those genes in the pathophysiology of EOO.

The alterations affecting glutamate receptors identified in two EOO patients were a partial gain of the gene encoding the ionotropic glutamate receptor *GRIK1* (Ob_33) and a gain partially encompassing the gene encoding the metabotropic glutamate receptor *GRM7* (Ob_22). L-glutamate is one of the main excitatory neurotransmitter in the CNS and activates both ionotropic and metabotropic glutamate receptors. A nonsense mutation was found in an additional patient in *GRIK1*. The metabotropic glutamate receptor 5 (mGluR5) plays a relevant role in energy balance and feeding. Adult mice lacking mGlu5 weighed significantly less than littermate controls and resisted diet-induced obesity [47]. Pharmacological approaches have described a reduction of food intake in response to antagonists of mGluR5 in a baboon model of binge-eating disorder [48] and in mGluR5+/+, but not mGluR5-/- mice [47]. On the contrary, dose-dependent stimulation of food intake has been described in rodents after injection of a mGluR5 agonist [49]. Moreover, the metabolic status and leptin can modify astrocyte-specific glutamate and glucose transporters, indicating that metabolic signals influence glutamatergic synaptic efficacy and glucose uptake [50]. Interestingly, *GRM7* is likely a loss of function intolerant gene given the difference between expected and observed frequency of loss of function variants in ExAC (25 expected, 1 observed). Partial gains, depending on the location, might act as loss of function alterations when disrupting the gene. Considering these data, glutamate receptors are promising candidates in the pathophysiology of obesity.

Several alterations affecting *GRPR* gene were identified, including a gain encompassing the whole gene and 4 point mutations (present in 5 subjects, two males and three females) while none were found in controls. The male patients with hemizygous *GRPR* RSVs had inherited the variant from heterozygous mothers. Both patients had very early onset obesity in infancy presenting a quite severe phenotype at diagnosis (+5SD and +9SD respectively). One of the mothers (patient Ob_162) had a BMI within the normal range while the other presented adult-onset obesity. Thus, the phenotype of both males is more severe than the phenotype of their mothers, consistent with X-linked inheritance. *GRPR* encodes the receptor of gastrin-releasing peptide. Gastrin is a hormone secreted by the gastric antrum and duodenum in response to gastric distension and the presence of food in the stomach. This hormone increases the production of hydrochloric acid, pepsinogen, pancreatic secretions and bile to facilitate food digestion and also promotes satiety [51]. It is a hormone directly implicated in the regulation of food ingestion and satiety and, thus, a candidate to be associated with obesity (directly or by an alteration of a gene included in the pathway, such as *GRPR*).

A possible recessive pattern of inheritance or a double hit mechanism was identified in a patient who harbors a deletion partially encompassing *SLCO4C1* and *SLCO6A1* (MIM 613365) and a RSV in *SLCO4C1*, each alteration inherited from one of the progenitors. Considering that the CNV was inherited from an obese progenitor and the RSV from the non-obese mother, we postulate a major contribution of the CNV and an additional but likely milder effect of the RSVs. The SLCO4C1 belongs to the organic anion transporter family and is involved in the membrane transport of thyroid hormones, among others. Interestingly, none homozygote subjects for loss of function variants has been described in ExAC.

Other rearrangements were found in single EOO patients, including those in regions previously associated to disease, such as 22q11.2 or 1q21.1. However, the evidence to link these genomic regions to obesity susceptibility is still weak and further data will be needed.

Except for the patient with a biallelic alteration in *SLCO4C1* and the *de novo* deletion, all CNVs and RSVs identified are heterozygous in the patients and inherited from one of the parents. Parents carrying the allele also showed an obese phenotype as well in most cases. Thus, a dominant effect (either hypo or hypermorphic) for these rare genetic variants with additive effects is suggested, leading to a more severe phenotype in the younger generation. This effect has also been found in other studies [52] and can be due to the more “obesogenic” environment that has developed in industrialized societies during the last two decades. Our results, along with previous genetic, family-based and epidemiologic studies, further indicate that EOO etiology is complex and mostly multifactorial, with the presence of some alleles that can behave as highly penetrant susceptibility variants or monogenic forms of obesity.

In summary, our findings reveal a higher burden of rare CNVs in patients with EOO compared to controls, including novel CNVs likely associated with familial obesity. Dosage sensitive genes altered by these CNVs are candidates for contributing to the pathogenesis of EOO. Some of these genes also harbor patient-specific RSVs, reinforcing their putative role in the pathophysiology of obesity. *NPY*, *GRPR*, *SLCO4C1* and glutamate receptors emerge as novel candidate genes involved in monogenic familial obesity.

## Materials and methods

### Subjects

Criteria for severe EOO was a BMI more than three standard deviation measures above the mean for age and gender with onset earlier than 3 years of age. All cases underwent a detailed clinical examination as well as family history in search of syndromic forms of obesity, which were discarded. All studies were performed as part of a research project approved by the Medical Ethical Committee of the Hospital Infantil Universitario Niño Jesús, after receiving written informed consent from the family.

Blood samples from patients were collected. Parental blood samples were also collected in cases in which an alteration was identified. DNA from patients and parents was isolated from total blood using the Gentra Puregene Blood kit (Qiagen) according to manufacturer’s instructions. We excluded genomic and epigenetic alterations associated with pseudohypoparathyroidism (MIM 103580), Prader-Willi, Temple (MIM 616222) and Beckwith-Wiedemann syndromes with a custom-made panel (S2 Table) of Methylation Specific Multiplex Ligation Dependent-Probe Amplification (MS-MLPA) [53].

A total of 480 unrelated subjects with severe EOO were included in the study. As controls for CNV and RSV association analyses, we studied 480 adult individuals of Spanish origin with a current BMI lower than 25 and no known history of childhood obesity, obtained from the National DNA Bank from the University of Salamanca (Spain).

### Molecular karyotyping

An initial sample of 157 probands was studied by using Omni1-Quad (64 subjects) or Omni Express SNP (93 subjects) platforms, Illumina. Copy number changes were identified using the PennCNV software with stringent filtering, as previously described [54]. CNVs encompassing known genes (RefSeq hg19), longer than 100 kb and with a frequency in control samples lower than 1/2,000 were selected. The frequency of each CNV in the control population was determined using 1M Illumina SNP array data of a total of 9,820 samples from two databases: 1) 8,329 individuals previously used as population controls for developmental anomalies [55] (81.2% of European descent, 2% African, and 16.5% other/mixed ancestry), and 2) 1,491 Spanish adult individuals from the Spanish Bladder Cancer/EPICURO study, which includes 1034 patients with urothelial cell carcinoma of the bladder and 457 hospital-based generally healthy controls with a mean age of 63.7 years [54]. To determine the frequency of CNV in the X chromosome, only the Spanish controls were considered, as data from the other cohort was not available. Given the size of the Spanish control sample (1,491), alterations in the X-chromosome absent in controls or only present in one subject were considered as rare. Briefly, a Hidden Markov Model (HMM) based on both allele frequencies and total intensity values was used to identify putative alterations, followed by manual inspection in conjunction with user guided merging of nearby (<1 Mbp between for arrays with <1 million probes and <200 kbp for arrays with >1 million probes) calls, which represent a single region broken up by the HMM, or gaps. All samples on arrays with densities <1M probes were filtered by a maximal genome-wide LogR ratio standard deviation of 0.25, while the high density 1.2 million probe WTCCC2 data was filtered using an increased standard deviation cut-off of 0.37. Mosaic alterations were excluded. For the two datasets where the Illumina array mapping corresponded to build35 (NHGRI), we utilized the autosomal calls generated previously [40] and mapped the coordinates to build36 using the UCSC LiftOver tool [56].

### Estimation of rare CNV burden

In order to compare the global burden of rare CNVs in patients and controls, data from 500 individuals randomly selected from the Spanish Bladder Cancer/EPICURO study and not included as controls for the CNVs frequency determination [54] were used. For the comparison, only CNVs in autosomal chromosomes with a minimum length of 100 kb, altering genes, and a frequency in control samples lower than 1/2,000 were considered (S3 Table). Alterations totally overlapping with segmental duplications were excluded to minimize biases due to the different probe coverage among microarray platforms.

### Multiplex Ligation-Dependent Probe Amplification (MLPA)

An MLPA assay was designed to validate genetic alterations detected by SNP platforms and to study inheritance in families (available upon request). A total of 100 ng of genomic DNA from each sample was subject to MLPA using specific synthetic probes (sequence available upon request) designed to target the specific CNV detected. All MLPA reactions were analyzed on an ABI PRISM 3100 Genetic analyzer according to manufacturers' instructions. Each MLPA signal was normalized and compared to the corresponding peak height obtained in control samples [57]. The MLPA assay was also used to analyze the frequency of the CNVs identified in the entire cohort (480 subjects) and in the control population (480 individuals).

### Targeted capture sequencing of pooled DNA for RSV identification

To study RSVs in the genes included in the CNVs, an enrichment kit was designed to capture all the coding regions of the selected genes (n = 14). The targeted enrichment was done with SeqCap EZ Choice Enrichment Kits (Roche Sequencing) and the massive sequencing with MiSeq (Illumina).

In order to sequence a high number of patients and controls (960 in total) in a cost-efficient manner, a pooled DNA approach was used [39]. Each sample was included in two different pools, and each pool contained 20 samples, avoiding two samples sharing both pools. A priori, any heterozygous RSV should be present in approximately 1 every 40 reads (2.5%). Thus, to ensure the identification of all RSVs (expected to be found in just one or few individuals) a high coverage was required.

To discriminate real variants from false positives due to extremely high coverage, we optimized the analysis pipeline. Variant calling was done with MuTect [58] to detect variants in a low proportion of reads. We also considered the quality of reads (base quality >15 in each pool) and the absence of strand bias (between 0.2 and 0.8) to define potential real variants from false positives.

To analyze the results and compare patients and controls, we focused on RSVs. We first established the frequency of each variant in the general control population using EXAC as the reference database, composed of 60,706 unrelated individuals sequenced as part of various disease-specific and population genetic studies. All alterations present in more than 1/1,000 alleles in EXAC in any of the populations included in the dataset were excluded. We specially focused on sequence changes with potential functional consequences, including loss of function variants (nonsense, frameshift and splice sites), missense variants predicted as pathogenic and changes in highly conserves residues. To search for recessive patterns of inheritance, we explored biallelic changes and RSVs that might act as second-hits in patients with previously identified CNVs.

### Sanger sequencing

To validate the RSVs detected by NGS and to define the segregation in each family, we designed primers to amplify an amplicon encompassing the variant and sequenced the amplicon by Sanger technology (available under request).

### Co-expression enrichment analyses

Using Genemania, we explored the co-expression between our candidate genes and the selected subset. To test if there was an enrichment of shared co-expressed partners, 500 sets of 15 genes with expression data available were randomly selected with Molbiotools (http://www.molbiotools.com/). For each set of genes the number of shared co-expressed partners was determined and compared with the interactions between our candidates and the set of obesity-related genes. The empirical p-value was calculated based on the fraction of shared co-expressed partners.

## Supporting information

S1 Table Description of mouse models with a phenotype related to body mass index caused by alterations in genes included in CNVs detected in the patients’ cohort. (XLSX)

S2 Table Probes included in the custom-made panel of Methylation Specific Multiplex Ligation Dependent-Probe Amplification (MS-MLPA) to exclude genomic and epigenetic alterations associated with pseudohypoparathyroidism, Prader-Willi, Temple and Beckwith-Wiedemann syndromes. (XLSX)

S3 Table CNVs in autosomal chromosomes with a minimum length of 100 kb, altering genes, and with a frequency in control samples lower than 1/2,000 detected in the control cohort. (XLSX)
